# Information Theory and Atrial Fibrillation (AF): A Review

**DOI:** 10.3389/fphys.2018.00957

**Published:** 2018-07-18

**Authors:** Dhani Dharmaprani, Lukah Dykes, Andrew D. McGavigan, Pawel Kuklik, Kenneth Pope, Anand N. Ganesan

**Affiliations:** ^1^College of Medicine and Public Health, Flinders University of South Australia, Adelaide, SA, Australia; ^2^Department of Cardiovascular Medicine, Flinders Medical Centre, Adelaide, SA, Australia; ^3^Department of Cardiology, University Medical Centre, Hamburg, Germany; ^4^College of Science and Engineering, Flinders University of South Australia, Adelaide, SA, Australia

**Keywords:** information theory, atrial fibrillation, entropy, AF mapping, catheter ablation

## Abstract

Atrial Fibrillation (AF) is the most common cardiac rhythm disorder seen in hospitals and in general practice, accounting for up to a third of arrhythmia related hospitalizations. Unfortunately, AF treatment is in practice complicated by the lack of understanding of the fundamental mechanisms underlying the arrhythmia, which makes detection of effective ablation targets particularly difficult. Various approaches to AF mapping have been explored in the hopes of better pinpointing these effective targets, such as Dominant Frequency (DF) analysis, complex fractionated electrograms (CFAE) and unipolar reconstruction (FIRM), but many of these methods have produced conflicting results or require further investigation. Exploration of AF using information theoretic-based approaches may have the potential to provide new insights into the complex system dynamics of AF, whilst also providing the benefit of being less reliant on empirically derived definitions in comparison to alternate mapping approaches. This work provides an overview of information theory and reviews its applications in AF analysis, with particular focus on AF mapping. The works discussed in this review demonstrate how understanding AF from a signal property perspective can provide new insights into the arrhythmic phenomena, which may have valuable clinical implications for AF mapping and ablation in the future.

## Introduction

Catheter ablation is a potentially curative treatment for atrial fibrillation (AF) that has been gaining interest within the last few decades. It uses percutaneously induced catheters to apply focal burns to specific areas of heart muscle in order to cease or modify the AF (Baumert et al., 2016). This method was first popularized following the breakthrough investigation published by Haissaguerre et al. (1998), which reported successful termination of up to 90% of paroxysmal AF (PAF) cases using catheter ablation on ectopic triggers located at the pulmonary veins. Although pulmonary vein isolation (PVI) is recognized as a landmark development for the treatment of paroxysmal AF, the extending application of ablative therapies for the highly varied persistent AF populace has seen relatively lower rates of success (Verma et al., 2015). Consequently, the optimal approach to ablation in this population is currently the subject of ongoing debate, as effective ablation targets remain unknown (Verma et al., 2015).

To help determine potentially effective targets, AF mapping is employed. The aim of AF mapping is to locate triggers and substrates that lead to AF termination, which in turn can be used as targets in clinical ablation procedures (Baumert et al., 2016). Unfortunately, the highly complex wave dynamics of AF have thus far been responsible for the difficulties in understanding what these effective targets may be, as the mechanisms underlying AF are arguably one of the most challenging problems in cardiology. As a result, a number of mapping approaches have been explored, including complex fractionated electrograms (CFAE) (Nademanee et al., 2004; Nademanee and Oketani, 2009; Hayward et al., 2011; Li et al., 2011), dominant frequency (DF) by Fast Fourier transform (Skanes et al., 1998; Sanders et al., 2006) and unipolar electrogram reconstruction (FIRM) (Narayan et al., 2012). Unfortunately, these approaches have either lead to conflicting outcomes (Nademanee et al., 2004; Narayan et al., 2012) or are still undergoing investigation (Dawes et al., 1992). In light of this, it is clear that exploration of new mapping methods are both warranted and necessary to understanding the mechanisms of AF and potentially improving the efficacy of catheter ablation.

Information theoretic-based approaches may be an appealing new avenue in AF mapping, as they have (i) a strong theoretical foundation in mathematics and (ii) use quantitative definitions rooted in intrinsic signal properties, instead of arbitrary, empirically derived definitions. In addition, although information theoretic approaches are seldom used in the field of cardiology, such techniques are already prevalent and widely accepted in other disciplines such as engineering, neurobiology, computer science, physics, quantum computing, linguistics, and cryptography (Verdu, 1998).

The objective of this review is to provide an overview of information theory in atrial fibrillation (AF). We begin by introducing the concept of information theory, and follow by discussing the use of information theoretic-based measures of entropy in three common areas of AF research: (i) AF detection, (ii) AF prediction and characterization, and (iii) AF mapping. Within the context of AF detection, studies successfully implementing entropy measures to distinguish between normal sinus patterns and AF are presented. Specifically, such studies develop AF detection algorithms based on detecting variations in the RR interval, or changes in the ECG morphology. Following this, the application of entropy for AF prediction and characterization is also described, outlining the use of entropy-based measures to understand the dynamical properties associated with the onset and termination of AF, as well as paroxysmal and persistent AF. Finally, we detail the current studies that employ information theoretic measures for AF mapping with respect to rotor identification, quantifying AF synchronization, and studying information flow within the atria. To conclude, we discuss the potential gaps in AF research that information theory may be able to address both now, and in the future.

## Information theory

### What is information theory?

Information theory is a branch of mathematics that incorporates probability theory and statistics (Ephremides, 2009). Modern information theory was established after the publication of Claude E. Shannon's seminal original paper (Shannon, 1948), which earned him the title of pioneer and founding father of information theory. Shannon's work introduced, for the first time, a number of key ideas that shaped the field of information theory, including the concept of digitizing information into binary digits known as “bits,” the formal architecture of communication systems, and source coding, which deals with the efficiency of data representation (Shannon, 1948). In short, the scope of information theory focuses on the transmission, processing, storage, and receiving of messages (Aftab et al., 2001; Lombardi et al., 2016). Although information theory was initially developed for use in communication systems, principally concerning itself with the transmission of telecommunication signals, it is now commonly used in a number of fields such as computer science, engineering, neuroscience and linguistics (Verdu, 1998; Xiong et al., 2017).

### What is information and how can it be measured?

As Shannon argued, the semantic aspects of communication can be thought of as irrelevant to the engineering problem (Shannon, 1948). Consequently, the term “information” in reference to information theory does not refer to the meaning of a message as one might assume intuitively, but instead how much can be learnt from that message (Lombardi et al., 2016). To conceptualize this further, take for example the scenario in which someone is asked to guess a number from 1 to 10, whilst obtaining help from a friend through clues. With respect to information theory, the clue itself does not matter, but the amount of information that can be inferred from the clue does. As such, if they are told that the number is >11, then this clue is deemed uninformative. On the other hand, if they are told that the number is even, then this fact is considered much more informative, though revealing that the number is odd would also be equally as informative, as these both reduce the possible selections to 5. In this respect, information can be thought of as ***how much*** is learnt, rather than ***what*** is learnt (Shannon, 1948).

Relating to this concept is the information theoretic measure known as “entropy.” As information can alternatively be thought of as the amount of uncertainty that is eliminated or resolved, measuring this uncertainty will intuitively quantify information. Conceptually speaking, entropy utilizes this principle to measure information content, with greater uncertainty in turn generating higher entropy (Shannon, 1948; Cover and Thomas, 1991; Gray, 2011). As entropy increases with uncertainty, it will be maximal for completely random systems (Shannon, 1948; Costa et al., 2002b). Such metrics have potentially useful clinical implications, particularly with respect to diagnostic tools using biological signals and understanding the underlying dynamic properties of physiological systems (Costa et al., 2005).

Although there are several ways that information can be measured outside of entropy, it is one of the most prevalently used classical measures of information theory, particularly in the context of AF. With this in mind, entropy-based approaches to quantifying information will be the focus of this review, and an overview of the various entropy algorithms commonly used to analyze AF will be described in the following.

## Shannon entropy

Named after Claude E. Shannon himself, Shannon entropy is the classical measure of information theory and measures the *Shannon information content* of a random variable (Shannon, 1948; Xiong et al., 2017). It was first introduced by Shannon to describe the relationships between information, noise and power in a digital communication stream (Aftab et al., 2001), quantifying the amount of storage required to store a signal (in bits) (Vajapeyam, 2014). Now, it is also commonly used as a measure of information content across many fields (Aftab et al., 2001). Shannon entropy (ShEn) can be defined as:

(1) $$ShEn=-\sum_{i\;=\;1}^{M}p(i)\log_{2}\;p(i)$$

where *M* is the number of discrete values the variable can take, and *p*(*i*) the probability density function of the variable *x* assuming the *i*^*th*^ value. Note that Shannon entropy is given in the unit bits (Shannon, 1948).

An intuitive example of how information is quantified using ShEn is the simple coin toss. A fair coin with a head and tail will result in maximum entropy, as the outcome cannot be predicted. As a result, the probability of choosing the correct outcome is $\frac{1}{2}$, as there are two possible outcomes that may occur with equal probability. Each coin toss will deliver one bit of information, as (Shannon, 1948):

(2) $$ShEn=-\sum_{i\;=\;1}^{2}\left(\frac{1}{2}\right)\log_{2}\left(\frac{1}{2}\right)=1\;bit$$

Conversely, a double-headed coin will result in an entropy of zero, as the probability of the outcome is 1/1. Hence there is no uncertainty, and no information is gained from the outcome of the coin toss (Shannon, 1948):

(3) $$ShEn=-\sum_{i\;=\;1}^{1}\left(1\right)\log_{2}\left(1\right)=0\;bits$$

**Figure 1:**
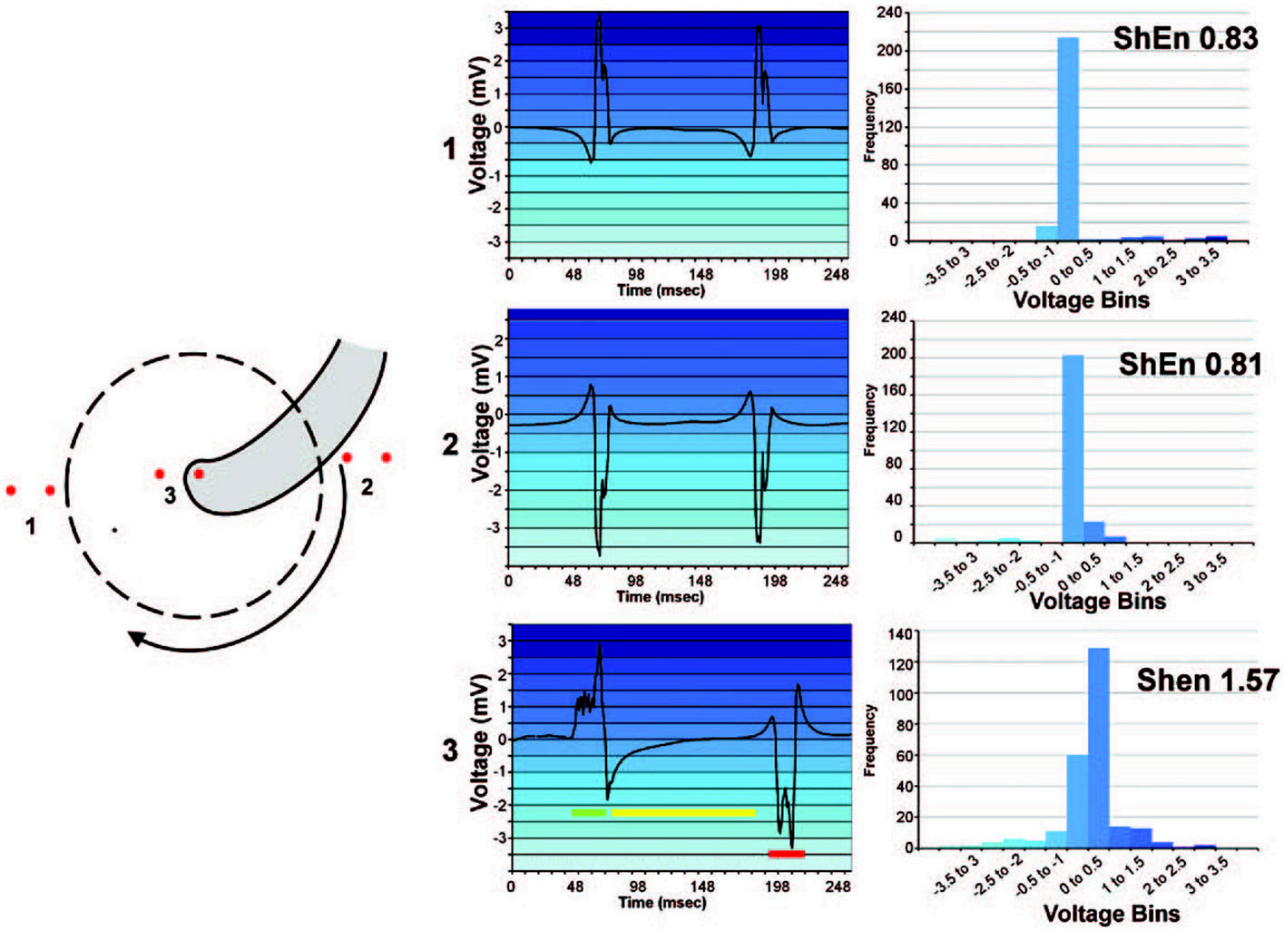
High ShEn correlates to the pivot of a spiral wave. An example of the relationship between ShEn and the amplitude distribution in a simple rotating wave. An amplitude distribution (histogram) is generated by binning samples of the signal in amplitude bins. Bipoles positioned in location 1 and 2 (at the periphery of the rotating wave) experience consistent activation direction, leading to more regular EGM morphology and a narrow amplitude histogram. Conversely, the bipole at position 3 (at the rotor pivot) experiences sharp local deflection (green), but secondary activity as the wavefront changes direction, including intermediate activity (yellow) and an inverted potential (red). Consequently, signal values are binned over a broader range of amplitudes, leading to higher ShEn. Reprinted from Ganesan et al. (2012) with permission.

In AF analysis, ShEn is often used to measure the information content of an ECG or EGM. Typically, this is achieved by constructing the amplitude distribution or histogram of the signal (Shannon, 1948; Ganesan et al., 2012, 2014; Baumert et al., 2016). Specifically, a voltage histogram can be acquired by binning signal samples according to its amplitude. Following this, the relative probability density function *p*(*i*) is obtained by dividing the sum of counts in each amplitude bin by the total number of counts. In effect, ECG or EGM with regular morphologies (i.e., signals that only possess a few states) will yield a narrow amplitude distribution (Ganesan et al., 2012). Conversely, complex morphologies containing a number of dissimilar deflections, such signals in AF, will lead to more varying amplitudes and in turn a broader amplitude distribution (Figure 1) (Ganesan et al., 2012). In effect, as ShEn is taken a sum of the probabilities, broader amplitude distributions will result in higher ShEn (Ganesan et al., 2012, 2014).

The ShEn equation defined in (1) can be classified as a “static” measure, as it does not consider any temporal information when describing the observed probability distribution. In other words, it measures information content by quantifying the amount of information contained only in the present value of the time series (Xiong et al., 2017).

## Approximate entropy

In contrast to the “static” measure of ShEn described in (1), “dynamic” measures of entropy are those that study the information content of a process representing the activity of a system that is changing over time. An example of such a dynamic measure of entropy is conditional entropy (Xiong et al., 2017).

Conditional entropy, also referred to as the Kolmogorov-Sinai entropy (Eckmann and Ruelle, 1985), is defined as the average rate of creation of new information. Generally speaking, the current state of an observed process is partly determined by its past, but also conveys some amount of new information that can't be inferred from the past. Conditional entropy measures this residual information to quantify the rate of creation of new information (Xiong et al., 2017). In mathematical notation, this can be given by:

(4) $$\begin{array}{l} C\left(X\right)=H\left(X_{n}|X_{n}^{-}\right)=\;H\left(X_{n}^{-},X_{n}\right)-H\left(X_{n}^{-}\right)\\ \;=\;-E\;[\log p\left(x_{n}|x_{1},\ldots ,\;x_{n-1}\right)] \end{array}$$

where *p*(*x*_*n*_|*x*_1_, …, *x*_*n*−1_) is the conditional probability that *X* assumes the value *x*_*n*_ at time *n*, given that previous values are taken at *x*_1_, …, *x*_*n*−1_ (Xiong et al., 2017). In effect, if the process is fully predictable, the system will not create new information and hence the conditional entropy is equal to zero. Contrastingly, a fully random process produces information at the maximum rate and will yield maximum conditional entropy. If the process is stationary, the system will produce information at a constant rate, and therefore the conditional entropy will not change over time (Xiong et al., 2017).

A number of entropy estimates and measures have been developed to quantify conditional entropy. One specific example, which is commonly used to study physiological signals, is Approximate Entropy (ApEn). ApEn is a regularity metric that was originally developed for physiological signals such as heart rate (Ganesan et al., 2014). As accurate entropy calculation using regularity statistics is often found unfeasible in real-life applications due to the influence of system noise and the large amounts of data required, Pincus developed ApEn to manage these limitations (Pincus et al., 1991; Ganesan et al., 2014). It can be noted that the approximate entropy family of statistics has been widely implemented in clinical cardiovascular studies (Pincus et al., 1991, 1993; Dawes et al., 1992; Fleisher et al., 1993, 1997; Goldberger et al., 1994; Ryan et al., 1994; Mäkikallio et al., 1996, 1998; Tulppo et al., 1996; Ho et al., 1997; Lipsitz et al., 1997; Hogue et al., 1998; Nelson et al., 1998; Palazzolo et al., 1998; Schuckers, 1998; Korpelainen et al., 1999).

Specifically, ApEn quantifies the amount of regularity in a signal by measuring the logarithmic likelihood that runs of patterns similar to one another will remain similar when incrementally compared (Richman and Moorman, 2000; Baumert et al., 2016). The prevalence of repetitive patterns in a signal is identified by forming a sequence of vectors using the time series data, and measuring the difference between them (Baumert et al., 2016). If the relative difference between any pair of corresponding measurements is less than the length of the pattern, the pattern is deemed similar (Pincus, 1991). In mathematical notation, this can be expressed using the equation:

(5) $$ApEn(S_{N,}m,r)=\ln\frac{Cm(r)}{Cm+1(r)}$$

where *m* is the pattern length, *r* is the similarity criterion or threshold, and *Cm*(*r*) the prevalence of patterns of length *m* in the sequence *S*_*N*_ (Pincus, 1991). ApEn is quantified in bits.

Conceptualizing ApEn further, take for example two time series, *t*_1_ and *t*_2_:

$$\begin{array}{l} t_{1}=(1,\;2,\;1,\;2,\;1,\;2,\;1,\;2,\;1,\;2\ldots )\\ t_{1}=(1,\;2,\;1,\;1,\;1,\;2,\;1,\;2,\;2,\;1\ldots ) \end{array}$$

**Figure 2:**
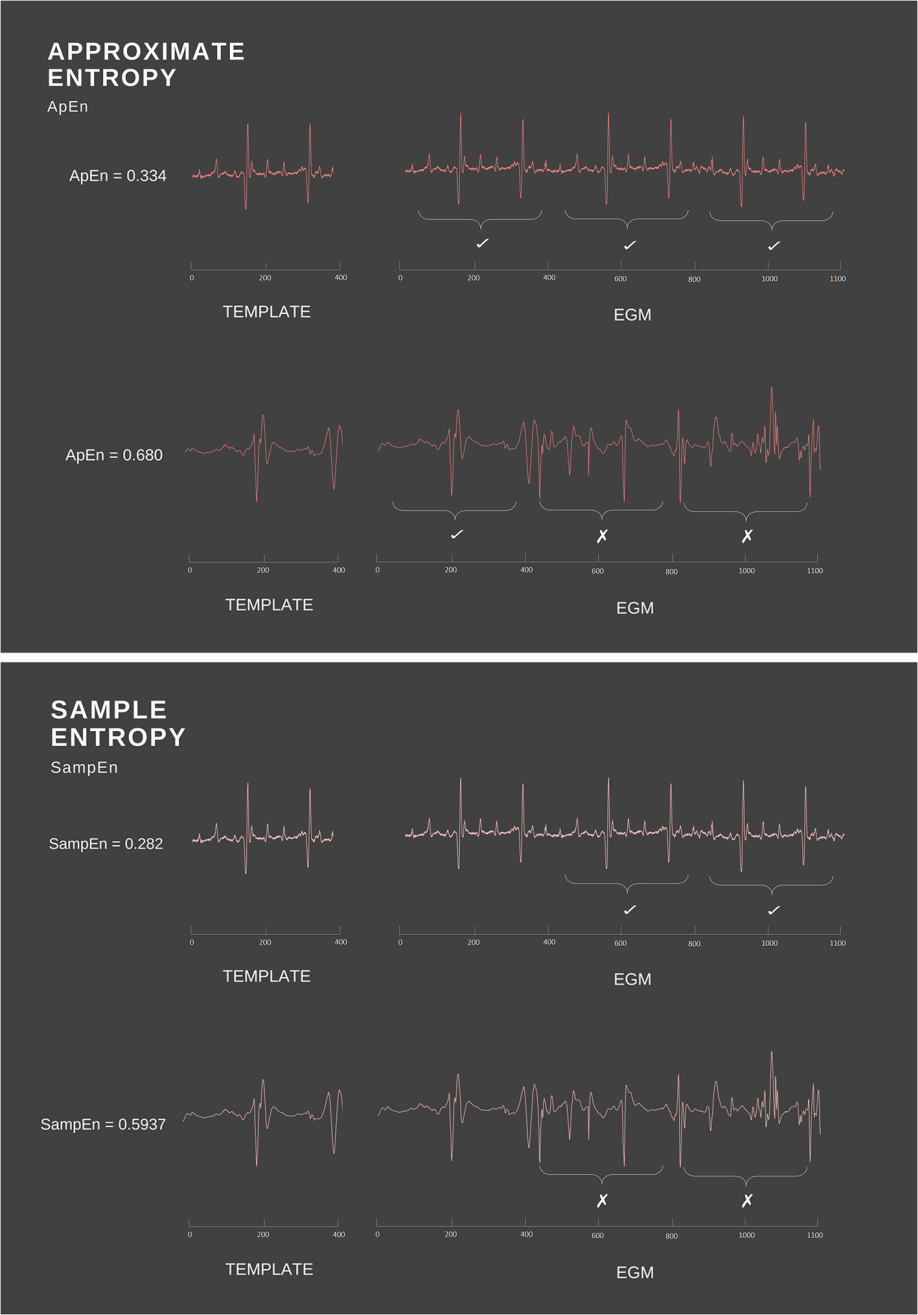
Simple example of ApEn and SampEn calculation. ApEn and SampEn examine the presence of repetitive patterns by measuring the difference between the template vector and the rest of the time series data. As ApEn will compare a vector to itself, the regularity of the overall signal is increased. In comparison, SampEn excludes self matches, thus avoiding biased regularity.

As *t*_1_ follows a very regular pattern alternating between 1 and 2 s, knowing that a term is valued at 1 will consequently allow the next value to be predicted, which in this case is always 2. Thus, *t*_1_ possesses high predictability and low conditional entropy. Conversely, *t*_2_ demonstrates a much more random pattern and hence will possess greater conditional entropy. Translating this to the cardiac space, a signal in normal sinus rhythm will exhibit periodicity and relatively uniform complexes, thus ApEn will detect the presence of similar patterns and identify this regularity (Figure 2). On the other hand, signals with complex morphologies will exhibit less regular patterns, hence yield higher ApEn (Pincus, 1991).

Computationally speaking, as opposed to the binning method used in ShEn algorithms, a kernel estimator is often used when calculating ApEn. Specifically, the kernel estimator is a model free approach of constructing a probability distribution of a variable, which centers kernel functions at each outcome of the variable and uses the estimated probabilities to compute appropriate entropy estimates (Xiong et al., 2017). The distance of each point in the time series to the reference point is weighted depending on the kernel function, and commonly, a Heaviside kernel function is used. The Heaviside kernel sets a threshold *r* as the weight for each point, which is equal to the width of the Heaviside kernel function and determines the precision of the density estimation (Xiong et al., 2017). A small *r* obtains a more detailed estimate, but requites more data points to ensure accuracy, whilst a larger *r* yields coarse probability estimates as too many points neighboring the reference will be included. With this in mind, *r* is typically set to a fraction of the data variance in practical applications, e.g., a function of the standard deviation of the dataset, to remove the dependence of entropy on the amplitude of the observed process (Xiong et al., 2017). Other estimators, such as linear and nearest neighbor estimators, are also available but are not as widely utilized in ApEn computation (Xiong et al., 2017).

Another important computational consideration is the length of the compared runs or window length *m*. The window length *m* allows the ApEn algorithm to search through the sequence *S*_*N*_ and measure the likelihood or prevalence, *Cm*(*r*), that runs of patterns similar for *m* observations remain close on the next incremental observation (Yentes et al., 2013). Although there is no established consensus for ApEn parameter selection, it is typically suggested that an *m* = 2 be used for clinical data. Though such a value is frequently used in literature and allows comparison of study results to other published findings, it is important to acknowledge that ApEn estimates depend strongly on the combination choice of *m, r* and the epoch length *N*. Hence, thoughtful consideration with respect to what *m* represents in a biological sense is necessary for each individual dataset (Yentes et al., 2013).

## Sample entropy

Another estimate of conditional entropy is sample entropy (SampEn). SampEn is a modified rendition of ApEn that works in much the same fashion (Richman and Moorman, 2000; Richman et al., 2004). Computationally, however, SampEn differs in two primary ways: (i) it is not length dependent and (ii) it does not include self-comparison of the template vector (Richman and Moorman, 2000). In ApEn calculations, the template vector (the vector being matched to) is compared to all others in the time signal, including itself. As a consequence, the probability of the vector *Cm*(*r*) will never equal zero (Richman and Moorman, 2000). As a result of this, the overall ApEn is lowered, since a self-match will create the appearance of increased regularity (Richman et al., 2004). By foregoing comparisons between a vector and itself (Figure 2), Richman et al. were able to create the SampEn family of statistics with an ability to avoid this biased regularity (Richman and Moorman, 2000). SampEn is derived from previous approaches established by Grassberger and Procaccia (1983), Ben-Mizrachi et al. (1984), Grassberger (1988) and Grassberger et al. (1991), and uses the natural logarithm of the conditional probability that two vectors that are similar at *m* points will remain similar at an incremental point (Richman and Moorman, 2000). Specifically, SampEn can be defined mathematically as:

(6) $$SampEn=-\log\frac{\sum A_{i}}{\sum B_{i}}=-\log\frac{A}{B}$$

where *A*_*i*_ is the number of matches of length *m*+1 with the *i*^*th*^ template, *B*_*i*_ the number of matches of length *m* with the *i*^*th*^ template and *SampEn* the entropy measured in bits (Richman et al., 2004).

Like ApEn, SampEn is also commonly computed using kernel estimators. Computationally, estimation of SampEn using kernel estimation is achieved using the conditional entropy Equation (7), which is implemented with the Heaviside kernel function and uses the maximum norm to compute distances (Xiong et al., 2017):

(7) $$C\left(X\right)=H\left(X_{n}|X_{n}^{m}\right)=\;-\ln\frac{\langle p(x_{n},x_{n}^{m})\rangle }{p(x_{n}^{m})}$$

Where $p(x_{n}^{m}$) is used to estimate the joint probability distributions *p*(*x*_*n*−1_, …, *x*_*n*−*m*_) and $p\left(x_{n},x_{n}^{m}\right)$ in the *m*-dimensional and (*m*+1)-dimensional spaces spanned by $X_{n}^{m}\;$and (${X_{n},\;X}_{n}^{m}$). Note that 〈. 〉 represents the averaging across patterns, and *K* represents the Heaviside kernel function:

(8) $$K=\Theta \;\left(\left\|x_{n}-\;x_{i}\right\|\right)=\;\{\begin{array}{c} 1,\;\left\|x_{n}-\;x_{i}\right\|\;\leq \;r\;\\ 0,\;\left\|x_{n}-\;x_{i}\right\|\;\leq \;r \end{array}$$

and *p*(*x*_*n*_) the kernel estimate of the probability distribution:

(9) $$p\left(x_{n}\right)=\;\frac{1}{N}\sum_{i=1}^{N}K(\left\|x_{n}-\;x_{i}\right\|)$$

where || . || is the maximum norm. Consequently, this kernel estimate of conditional entropy reduces the bias seen in ApEn (Xiong et al., 2017).

In studies, SampEn demonstrated greater robustness over a broad range of conditions, which potentially makes it a more useful algorithm in studies analyzing physiological data (Richman and Moorman, 2000). SampEn also showed greater performance with short datasets, showing less dependency on the data length in comparison to ApEn estimates (Yentes et al., 2013). Like ApEn however, SampEn is also sensitive to parameter choice, though showed greater relative consistency over a broad range of possible combination values for *r, m*, and *N* (Costa et al., 2005). Despite this, care should still be taken when choosing SampEn parameters.

## Multiscale entropy

As discussed, ApEn and SampEn approaches evaluate the appearance of repetitive patterns to compute the regularity of a signal and calculate entropy. One potential limitation of these methods, however, is that increased entropy may not always translate to increased dynamical complexity (Costa et al., 2002b). As Costa et al. argue, entropy-based measures such as the Kolmogorov complexity and entropy rate, grow monotonically with randomness (Costa et al., 2005). Consequently, such measures will yield high entropies for uncorrelated random signals such as white nose, which possess low predictability but are not structurally “complex.” A randomized time series will also yield higher entropy than the original signal, despite the fact that the process of creating surrogate data destroys correlations and degrades the information content of the time series (Costa et al., 2002b). With this in mind, Costa et al. aimed to develop a quantitative measure of dynamical complexity with three basic hypotheses in mind: (i) that the complexity of a biological system reflects its ability to function and adapt in an evolving environment; (ii) biological systems operate between multiple spatial and temporal scales, thus possessing multiscaled complexity; and (iii) a number of disease states that reduce the adaptive capacity of the individual seemingly degrades the information carried by output variables (Costa et al., 2005). As such, Costa et al. introduce a multiscaled entropy (MSE) approach that quantifies entropy over a range of time scales (Humeau-Heurtier, 2015).

Using MSE, measurements have the ability to reflect that both completely ordered and completely random signals are not truly complex, and identifies that correlated random signals are more complex than uncorrelated random signals (Costa et al., 2002a, 2005; Costa and Healey, 2003). The inclusion of measurements from a variety of temporal scales also includes two major advantages: (i) the ability to assess complexity at longer and shorter time scales and (ii) quantification of the overall system complexity, which is equal to the sum of entropy values over all temporal scales (Busa and van Emmerik, 2016).

Computationally, MSE implements the SampEn algorithm to assess complexity. The primary motive for using SampEn as opposed to ApEn is its greater consistency over a broad range of *r, m*, and *N* values, as well as its reduced dependency on the time series length (Costa et al., 2005). In comparison to the multiscaled complexity approach introduced by Zhang et al. for physical systems (Zhang, 1991), which is based on ShEn, the use of SampEn also allows MSE to become better suited to physiologic time series'. The use of Shannon's definition of entropy in Zhang's method requires a large amount of virtually noiseless data in order to accurately map to a discrete symbolic sequence, which introduces limitations when applied to free-running physiologic signals (Costa et al., 2005).

In recognizing these considerations, Costa bases MSE on a modification of Zhang's and Pincus' approaches. Specifically, MSE comprises of two steps: (i) “coarse-graining” of the time series, which derives the representations of a system's dynamics at varying temporal scales (a form of resampling) and (ii) application of SampEn on each of the coarse-grained time series (Costa et al., 2005). Specifically, construction of a coarse-grained time series involves averaging a successively increasing number of data points using non-overlapping windows. Mathematically, each element of the coarse-grained signal is computed using:

(10) $$y_{j}^{\left(\tau \right)}=\;1/\tau \sum_{i=\left(j-1\right)\tau +1}^{j\tau }x_{i}$$

where τ represents the scale factor and 1 ≤ *j* ≤ *N*/τ (Costa et al., 2002a). In effect, the length of each coarse grained data will equal the length of the original time series divided by the scaling factor τ (Wu et al., 2013).

Following this, SampEn is used to calculate an entropy estimate for each coarse-grained time series plotted as a function of the scaling factor τ. This step is referred to as the multiscale entropy analysis. In traditional uses of SampEn, data from certain pathologic time series that may be chaotic/unpredictable but arise from less physiologically complex systems and mechanisms, such as data from episodes of atrial fibrillation, will result in higher SampEn. This is because such SampEn estimates are based on a single scale and hence will not account for features related to structure and organization on other scales (Costa et al., 2005). The MSE results published by Costa et al. support this, showing that at a single scale, the entropy assigned to the time series of atrial fibrillation and congestive heart failure patients is higher than those of healthy patients. Contrastingly, when analyzed at multiple scales, the time series of healthy subjects are assigned with highest entropy, reflecting that healthy cardiac dynamics are the most physiologically complex (Costa et al., 2005). Whilst these results contradict those obtained using traditional ShEn, SampEn, and ApEn algorithms, they more accurately reflect the physiological complexity of the underlying system.

It is important to note that although Costa's MSE algorithm is widely used in multiple fields, this approach still suffers from limitations. First, spurious MSE oscillations are introduced due to the inefficient process of eliminating fast temporal scales, and the original coarse graining procedure also artificially reduces MSE (Valencia et al., 2009). To rectify these issues, Valencia et al. develop a refined multiscale entropy approach (RMSE) (Valencia et al., 2009). The RSME approach utilizes a low-pass Butterworth filter instead of an FIR filter to eliminate fast temporal scales, which ensures a more accurate elimination of components above the specified cut-off frequency. In addition, RMSE uses a refined coarse graining procedure that implements a continuously updating *r*, defined as a percentage of the standard deviation of the filtered series. In effect, this compensates for the decrease in variance related to the filtering procedure for removal of the fast temporal scales (Valencia et al., 2009).

Though RSME overcomes spurious MSE oscillations and biased reduction of MSE estimates, it is difficult to reliably compute over short time series. In response, Faes et al. introduce the linear MSE (LMSE) method (Faes et al., 2017), which utilizes linear state-space models to provide a multiscale parametric representation of an autoregressive process observed at multiple time scales. LMSE exploits the state-space parameters to quantify the complexity of the process. Results show that in comparison to both RSME and MSE, application of LMSE to short cardiovascular data provides a better description of the physiological mechanisms producing biological oscillations at different temporal scales (Faes et al., 2017).

Another limitation of MSE is that the statistical reliability of SampEn for a coarse-grained time series is reduced as the time scale factor τ increases. This is because for an *N* point time series, the length of the coarse-gained series at scale factor τ is *N*/τ. Consequently, the larger the scaling factor, the shorter the coarse-gained series and hence, the variance of the estimated entropy will increase as the scaling factor increases. To overcome this, Wu et al. developed the concept of a composite multiscale entropy (CSME) to reduce the variance of estimated entropy values at large scales (Wu et al., 2013). Specifically, CSME achieves this by calculating the sample entropy of all coarse-gained time series and finding the mean of the τ entropy values, rather than only the first coarse-grained time series as proposed by Costa (Wu et al., 2013).

## Wavelet entropy

While the aforementioned algorithms all compute entropy in the time domain, entropy estimates can also be calculated in the frequency space. Broadly speaking, computing entropy in the frequency domain consists of transforming the time series using methods such as the Fourier transform or wavelet decomposition (Rosso et al., 2001). Although signals used within the medical field are predominantly presented in the time domain, representation in the frequency domain may provide advantages in certain applications. For example, studies have postulated that the frequency band with the highest strength correlates to the cycle length derived from time domain analysis (Ng et al., 2006), which is annotated as the dominant frequency (DF) (Sanders et al., 2005, 2006). This becomes potentially useful in signals with deflection, varying amplitudes, and more complex temporal patterns, wherein time domain measurements of the cycle length are likely to be inaccurate (Traykov et al., 2012). As this is often the case during AF, frequency-based analyses may provide a better measurement of the atrial rate (Traykov et al., 2012). It should be noted, however, that the frequency spectrum may also be determined by other factors outside of cycle length, such as morphology and amplitude (Ng et al., 2006; Elvan et al., 2009). As such, wavelet entropy methods that combine both entropy and frequency analysis may provide additional insights and more robust analyses in comparison to DF analysis alone (Ng et al., 2006; Elvan et al., 2009).

Specifically, wavelet entropy (WE) combines entropy and wavelet decomposition to provide an estimate of the degree of disorder present within a signal (Rosso et al., 2001). The wavelet entropy of a signal can be given by:

(11) $$WE=\;-\sum_{j\;=\;1}^{N}E_{j}\log(E_{j})$$

Where *E*_*j*_ is the relative energy associated with the wavelet coefficient at scale *j* and *N* the number of wavelet decomposition levels. Calculating entropy in this way provides a measurement of the amount of order or disorder in a signal, wherein WE will assume a value that is very low and close to zero for an extremely organized signal such as a periodic mono-frequency event, and high WE for random signals such as white noise (Ródenas et al., 2015). Consequently, EGM or ECG signals with greater complexity and irregularity will result in high WE.

## Transfer entropy

Transfer entropy (TE) is an information theoretic measure that can be used to understand the information transfer between joint processes (Barnett et al., 2009). In systems consisting of more than one component or variable, understanding information transfer between these variables can be extremely useful in determining its structure and mechanism. Many studies have attempted to study such relationships using an alternate information-theoretic measure known as mutual information (MI), which provides a model-free approach to quantifying information overlap between two variables (Vicente et al., 2011). Specifically, this is achieved by measuring the amount of information that can be learnt from one random variable by observing another. Unfortunately, MI measures do not capture dynamical and directional information exchange, and hence poorly describe causal relationships (Schreiber, 2000). For example, MI is symmetric under the exchange of signals and cannot differentiate between response and driver systems. Secondly, MI captures only the amount of information that is shared by two signals, rather than the information being exchanged (which better relates to causal dependence) (Schreiber, 2000). To provide an asymmetric measure, delayed MI, which measures MI between a signal and another lagged signal, has been proposed. Though delayed MI reflects certain dynamical structures as a result of the time lag, it is still flawed and can cause issues when shared information from a common input or history is present (Schreiber, 2000). To address these problems, Schreiber et al. develop transfer entropy (TE) to provide an alternative information theoretic measure that shares some of the desired properties of mutual information, but also considers the dynamics and directionality of information transfer (Schreiber, 2000).

To measure TE between two variables *X* and *Y*, one needs to measure the amount of uncertainty (entropy) that is reduced in future values of *Y* by knowing past values of *X*, given the past values of *Y*. In mathematical notation, the TE between *X* and *Y* can be given by:

(12) $$TE\left(\;X\to Y\right)=\;\sum_{y_{t+1,}y_{t}^{n},\;x_{t}^{m}}p(y_{t+1,}y_{t}^{n},x_{t}^{m})\log\frac{p(y_{t+1}|y_{t}^{n},x_{t}^{m})}{p(y_{t+1}|y_{t}^{n})}$$

where $x_{t}^{m}$ = (*x*_*t*_, …, *x*_*t*−*m*+1_), $y_{t}^{n}$ = (*y*_*t*_, …, *y*_*t*−*n*+1_), and m and n the orders of the Markov processes *X* and *Y* respectively (Vicente et al., 2011). Typically, TE estimations use the Shannon entropy algorithm during computation to provide a measure of the uncertainty between *X* and *Y* (Schreiber, 2000).

It can be noted that the concept of transfer entropy shares some overlap with the statistical notion of causal influence termed *Granger causality* (GC) (Barnett et al., 2009), which uses prediction via vector auto-regression to measure causality. Specifically, given sets of inter-dependent variables *X* and *Y*, Granger causality will say *X* Granger-causes *Y* if *Y* assists in predicting the future of *X*, beyond what *X* already predicts about its own future (Barnett et al., 2009). In contrast, the information theoretic notion of transfer entropy is framed in the context of *resolving uncertainty*. For example, it can be said that the transfer entropy from *Y* to *X* is the degree to which *Y* disambiguates the future of *X*, beyond what *X* already disambiguates about its own future (Barnett et al., 2009). This relationship was explicitly explored and detailed by Barnett et al. (2009), who shows that under Gaussian assumptions the concept of Granger Causality and Transfer entropy are in fact equivalent.

Although TE is prevalently used in neuroscience to understand the causal relationships between parts of the brain and responses to stimuli (Vicente et al., 2011), it has thus far received little attention in the cardiac space.

### Limitations of entropy

Although entropy is widely used in many fields, reliable estimation of information-theoretic quantities from empirical data can prove difficult. Firstly, the sensitivity of entropy estimates with respect to parameter selection can be problematic. Discretization of a time series using bins as commonly done in ShEn algorithms can pose potential problems, as inappropriately selecting bin-widths can lead to greater bias and reduce the accuracy of entropy estimates (Purwani et al., 2017).

Additionally, both ApEn and SampEn show significant 2-way and 3-way interactions between *m, r*, and *N*, hence are influenced heavily by the combination choice of these parameters (Yentes et al., 2013). Appropriate parameter selection is particularly critical when analyzing short data sets, as ensuring there is a sufficient number of matches can be problematic (Lake and Moorman, 2010). This can be seen in rapid diagnosis of AF. In such cases, choosing an *m* that is too large or *r* that is too small will result in too little template matches to estimate the conditional probabilities accurately. Conversely, if *m* is too small and *r* too large, all templates will match each other and cardiac different rhythms cannot be discerned (Lake and Moorman, 2010). Although SampEn is relatively more stable across varying data lengths in comparison to ApEn, inappropriate parameter selection, particularly in the choice of *r*, can still lead to inaccurate entropy estimates (Lake and Moorman, 2010).

In addition, although bipolar EGM entropy-based approaches of AF mapping (such as ShEn mapping) have been proposed for identification of rotational activity, problems may be encountered in areas where wave propagation dynamics mimic such rotational sources. For example, areas in which multiple waves precess and cross propagate in varying directions may instead appear to originate from a single rotational source and hence, result in high entropy estimates similar to those seen for rotors. This could potentially present problems for a targeted entropy guided ablation strategy in the future.

## Information theory and the intracardiac electrogram

**Figure 3:**
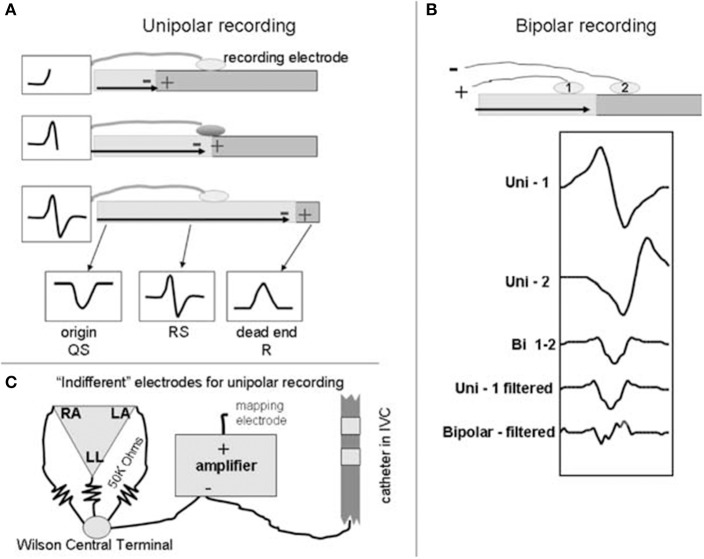
Basic generation of bipolar and unipolar recordings. A schematic to illustrate the basic generation of bipolar and unipolar electrograms. At the top of **(A,B)**, a horizontal bar is used to represent a sheet of myocardium with depolarization propagating from left to right. Example electrograms are shown in boxes. **(A)** Unipolar recording: the wavefront propagates toward the electrode, creating a positive deflection, creating an R wave. As the wavefront propagates past the recording electrode, an S wave is detected and hence creates the RS complex (middle schematic). Recording at the right side of the tissue (dead end) creates a monophasic R wave. **(B)** Bipolar recording: electrodes 1 and 2 are connected to the positive and negative inputs of the amplifier respectively. Electrograms generated via mathematical simulation are shown below the schematic. Compared to the signal from electrode 1 (Uni-1), the signal from electrode 2 (Uni-2) is slightly delayed (due to the wavefront reaching it later) and is inverted due to its connection to the negative input of the amplifier. Addition of the two signals generates the bipolar signal (Bi 1-2) that removes far field signal. Differentiating the signal (Unipolar- filtered) decreases far field component and produces a signal similar to the bipolar electrogram but slightly time shifted. Differentiating the bipolar signal (Bipolar-filtered) produces additional deflections and further complicates the signal. Indifferent electrode configurations are shown in **(C)**. Reprinted from Tedrow and Stevenson (2011) with permission.

To further discuss how information theory can be used in the context of AF mapping, we will first discuss the intracardiac electrogram. An intracardiac electrogram (EGM) is acquired by measuring the voltage difference between two recording electrodes (Tedrow and Stevenson, 2011). In the unipolar electrogram configuration, the anodal (positive) input of the amplifier is by convention connected to the exploring electrode, which is usually located at the tip of the catheter and in physical contact with the cardiac tissue (Tedrow and Stevenson, 2011; Baumert et al., 2016). Another intermediate electrode, often referred to as the reference electrode, that is distant from the heart is connected to the cathodal (negative) input of the amplifier (Tedrow and Stevenson, 2011; Baumert et al., 2016). As a product of this configuration, the resultant unipolar signal possesses a characteristic morphology due to the passing of planar wavefronts toward the recording electrode (Figure 3) (Tedrow and Stevenson, 2011). A distinctive biphasic complex is created as the wavefront reaches and passes the electrode, causing the wavefront deflection to become steeply negative (Baumert et al., 2016). This generates an RS complex (Tedrow and Stevenson, 2011). One limitation of the unipolar EGM is its vulnerability to far field activity (electrical activity from other parts of the cardiac chamber), electromagnetic interference (mains noise) and in the case of AF mapping, interference from ventricular depolarization (Tedrow and Stevenson, 2011; Baumert et al., 2016). Few studies have utilized unipolar EGM for mapping, however, these issues have largely hindered their use within research (Konings et al., 1994, 1997; Allessie et al., 2010; Tedrow and Stevenson, 2011).

In contrast, bipolar electrograms are obtained via the subtraction of two unipolar EGMs recorded proximally, which is typically achieved by subtracting from adjacent poles of the catheter (Baumert et al., 2016). Due to this configuration, much of the ventricular contribution to the signal is largely eliminated, and as such, bipolar EGM are generally preferred in clinical settings (Tedrow and Stevenson, 2011; Baumert et al., 2016). Unfortunately, however, the timing of local activations is less defined in comparison to unipolar EGM, though in homogeneous tissue the initial peak coincides with local depolarization time (van der Does and de Groot, 2017). In AF, bipolar EGM morphologies are generally irregular and complex in comparison to their sinus rhythm counterpart, possessing many deflections (Baumert et al., 2016). EGM morphologies can be categorized into three types, as previously described by Wells et al. (1978) (Table 1).

**Table 1:** Characterization of Atrial Fibrillation (AF) in Man as defined by Wells et al. (1978).

**AF Type**	**Description**
Type 1	Discrete atrial complexes with varying morphological appearance but with discrete isoelectric baseline
Type 2	Discrete beat-to-beat atrial complexes but no isoelectric baseline
Type 3	Complex and highly irregular atrial EGMs with no discrete complexes or isoelectric intervals

*Characterization of AF into three AF types, which uses the bipolar EGM morphology during AF to provide classification*.

Relating to the EGM morphology is the previously discussed concept of entropy. When applied to the intracardiac electrogram, entropy approaches have the potential to provide clinical insights into the underlying dynamics of AF. As entropy is linked to the information content of a signal, EGM with complex, non-uniform morphology will result in greater uncertainty and higher entropy. Conversely, an EGM with a regular, periodic morphology will result in lower entropy (Ganesan et al., 2012). This characteristic makes entropy particularly useful in AF applications in which the ability to distinguish between AF and non-AF signals is required (AF detection algorithms), and where changes in the EGM morphology are thought to correlate to important AF triggers or substrates (AF mapping). The identification and localization of rotors provides a particularly interesting application of entropy within the AF mapping space, as it has been demonstrated that the pivot zone of a rotor experiences greater spatial uncertainty of wavefront direction, resulting in less stable bipolar EGM morphologies that can be quantified by entropy (Ganesan et al., 2012, 2014; Arunachalam et al., 2015). Other wave propagation dynamics are less well explored in relation to entropy, though additional mechanisms of interest (such as complex wavelet interaction regions) may also yield useful entropy characteristics. If statistical information theoretic approaches such as entropy can be used to pinpoint such potentially important AF landmarks, a targeted ablation strategy may become possible in the future.

In some ways, the concept of entropy shares some conceptual overlap with the notion of fractionation. Broadly speaking, the term fractionation is used to describe EGMs that possess multiple deflections and are prolonged (van der Does and de Groot, 2017), although no precise consensus definition currently exists (Baumert et al., 2016). As such, like entropy, CFAE essentially aims to provide some definition to describe the complexity of an electrogram. A number of sources are said to be responsible for fractionation, with the local collision of multiple wavelets, local re-entry and zones of slow conduction said to result in EGM fractionation during AF (de Bakker and Wittkampf, 2010; Waks and Josephson, 2014). Consequently, it is thought that there is some relationship between CFAE and the maintenance of AF (Waks and Josephson, 2014), thus CFAE mapping and CFAE guided ablation have previously been explored (Nademanee et al., 2004; Nademanee and Oketani, 2009; Berenfeld and Jalife, 2011; Hayward et al., 2011; Li et al., 2011; Chen et al., 2014). Though both CFAE and entropy aim to capture the qualitative property of signal fractionation to some degree, entropy is differentiated from CFAE by having a quantitative definition rooted in signal processing and mathematics, and does not use empirically derived definitions.

Outside of fractionation, entropy-based analysis of the EGM also has the potential to provide insights about the complex wave propagation dynamics underlying AF (Kośna et al., 2015). For example, transfer entropy may possess the ability to determine information flow during AF and in turn, uncover causality and electrophysiological pathways between various regions of the heart that may be involved during AF propagation. Consequently, analysis of information flow may be useful for identifying the atrial regions central to maintaining AF (Kośna et al., 2015).

## Current studies using information theoretic approaches in atrial fibrillation

Though information theoretic and entropy-based approaches remain relatively limited within atrial fibrillation research, a handful of studies have explored their use. Broadly speaking, the use of entropy in AF can be categorized into three groups, namely: (i) entropy for AF detection, (ii) entropy for AF characteristic determination, and lastly (iii) entropy for AF mapping. Some of the approaches using entropy in the current literature will be broadly discussed in the following, with particular focus on the role of entropy in AF mapping.

### Entropy for AF detection

Presently, with respect to the study of atrial fibrillation (AF), entropy is most widely used for the detection of AF in ECG recordings and Holter monitors. As AF episodes occur paroxysmally in the majority of patients, human-based diagnosis of AF can oftentimes be difficult and time consuming, hence automated and computerized methods of AF detection have become a lucrative diagnostic application of entropy (Ródenas et al., 2015; Cui et al., 2017).

Many algorithms have been developed to detect AF, which can often be broadly categorized as being based on either (i) P-wave detection or (ii) RR interval (RRI) variability (Lee et al., 2011). Of these two methods, AF detection using the temporal variability of the RR interval has become a much more common approach in literature as analysis of the P-wave morphology is often difficult, as ECGs can be noisy and are prone to motion artifact. In addition, the determination of a P-wave fiducial point is challenging due to its low amplitude during AF which makes it more susceptible to corruption through noise, in turn lowering the signal-to-noise ratio (Dash et al., 2009; Lee et al., 2011; Ródenas et al., 2015).

Various studies have used Shannon entropy (ShEn) in conjunction with various other measures of complexity such as the Turning Points Ratio (TPR) (Dash et al., 2009), Root Mean Square (RMS) of successive RR differences (Dash et al., 2009), symbolic dynamics (Zhou et al., 2014), and time-varying coherence functions (TVCF) (Lee et al., 2011), among others, to better capture the randomness in the signal and detect variability of the RRI time series (Dash et al., 2009; Lee et al., 2011; Zhou et al., 2014). As ShEn can be used to measure the level of uncertainty and information size in the signal, it can reflect whether the ECG morphology exhibits irregularities, and hence variability in the RRI time series. Results using these methods demonstrate high rates of sensitivity and specificity upwards of 95% (Dash et al., 2009; Lee et al., 2011; Zhou et al., 2014), indicating the feasibility of entropy for detecting RRI variability.

**Figure 4:**
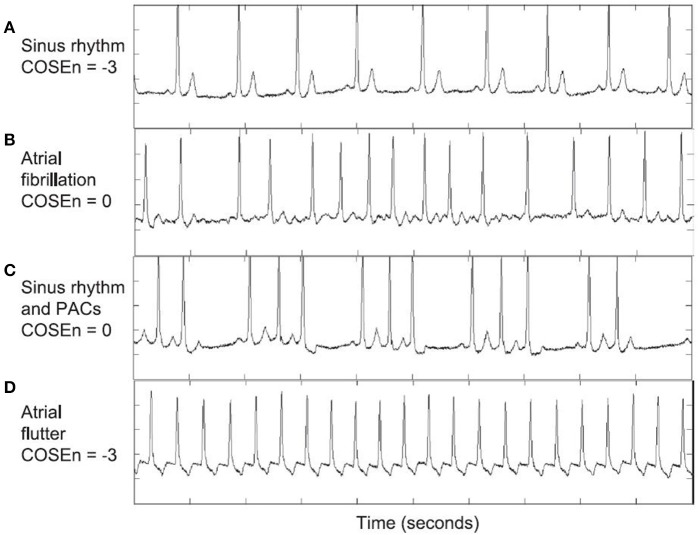
AF detection using COSEn (Coefficient of sample entropy). CoSEn values for ECGs from the MIT-BIH atrial fibrillation database used by Lake and Moorman (2010) to test automated AF detection using SampEn. Although CoSEn was able to differentiate between normal sinus rhythm **(A)** and atrial fibrillation **(B)**, sinus rhythm with ectopy **(C)**, and atrial flutter (AFL) **(D)** resulted in misclassification. This was due to (i) irregular RR intervals for sinus with ectopy, which increased SampEn and mimicked AF values for CoSEn and (ii) 2:1 AFL creating regular RR intervals, mimicking sinus values for CoSEn. Reprinted from Lake and Moorman (2010) with permission.

Although the RRI time series approach is frequently used, widely available and capable of providing adequate AF detection in a number of cases, entropy-based approaches have also been used independently to detect AF (Lake and Moorman, 2010; Carrara et al., 2015a,b). Sample Entropy (SampEn) algorithms have been used to detect the probability that runs of AF will match with others within the time series (Richman and Moorman, 2000; Lake and Moorman, 2010) (Figure 4). A benefit of SampEn is its ability to use short runs or bursts of AF as a template for matching, hence avoiding issues relating to short AF episode durations that are common with RRI variability-based methods. These studies showed that SampEn provided a high degree of accuracy in distinguishing AF from sinus rhythm (~95%), but encountered errors when atrial or ventricular ectopy were present, as this increased the entropy of the signal (Lake and Moorman, 2010; Carrara et al., 2015a,b).

The use of wavelet entropy (WE) for AF detection has also been explored in single lead electrograms (Ródenas et al., 2015). This method was used under the premise that using entropy and wavelet decomposition in conjunction increases the robustness of the detection algorithm to noise, artifacts and non-stationarities (Ródenas et al., 2015). Ródenas et al. use this method to calculate the WE on a TQ interval to identify the presence and absence of the P-wave in each beat of the ECG, which in turn determines the presence of AF. Results demonstrated a discriminant ability of approximately 95%, which is comparable to results from other studies (Lake and Moorman, 2010; Carrara et al., 2015a,b).

### Entropy for AF prediction and characteristic determination

To effectively treat atrial fibrillation, an understanding of the arrhythmia itself is also crucial. As such, another application of entropy is its use in determining the various characteristics of AF. One such area that has been studied are the changes in the RR interval dynamics preceding the onset of postoperative AF, as studying these characteristics may enable prediction of postoperative AF episodes. It has been hypothesized that heart rate variability (HRV), which can be used as an indicator of cardiac sympathovagal balance, would alter before the onset of postoperative AF and could be measured using entropy algorithms (Hogue et al., 1998). Findings on this have been conflicting, however, with Hogue et al. showing a decrease in ApEn upon the onset of AF (Hogue et al., 1998), whilst other studies show entropy and HRV analyses provide little predictive value when studying the onset of postoperative AF (Amar et al., 2003; Chamchad et al., 2011).

Adding to this, it is argued that the ability to predict the spontaneous onset of AF for non-postoperative patients is also important as it may allow prevention using electrical stabilization and various pacing techniques (Tuzcu et al., 2006). A number of studies have used ApEn and SampEn to predict the onset of paroxysmal AF (PAF), as these measures have the ability to measure the regularity of the time series signal, and hence quantify the heart rate variability (HRV) (Vikman et al., 1999; Amar et al., 2003; Shin et al., 2006; Tuzcu et al., 2006). Findings showed that ApEn and SampEn could predict the onset of AF as entropy of the HRV reduced significantly in ECG preceding AF, in comparison to those distant from an AF episode (Vikman et al., 1999; Shin et al., 2006; Tuzcu et al., 2006).

**Figure 5:**
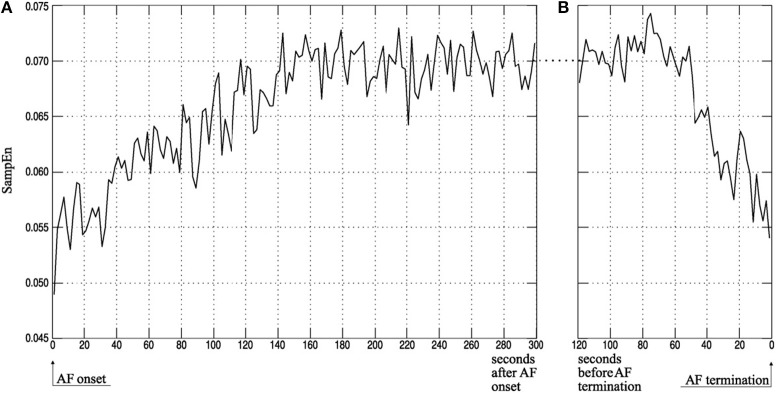
Atrial activity organization time course of ECG recordings measured using SampEn. Average atrial activity (AA) organization, quantified using SampEn, for **(A)** the first 5 min after AF onset and **(B)** the last 2 min before AF termination as computed by Alcaraz and Rieta (2010). Findings show that SampEn, and in turn the AA organization, decreases in the first few minutes of AF onset and increases within the last minute of spontaneous AF termination. Reprinted from Alcaraz and Rieta (2010) with permission.

In the same breath, predicting the termination of PAF may also have clinical implications, as it may in turn help improve management of the arrhythmia and avoid unnecessary treatments (Alcaraz and Rieta, 2009a). Specifically, SampEn has been used to study the atrial activity (AA) organization from surface electrocardiograms (ECG) and predict the spontaneous termination of AF. It has been shown that SampEn of terminating AF episodes are lower in comparison to non-terminating episodes (Alcaraz and Rieta, 2009a,b, 2010) (Figure 5).

Lastly, analysis of the HRV complexity using SampEn have also been used to evaluate the characteristics of both PAF and persistent AF (Sungnoon et al., 2012). The study conducted by Sungnoon et al. aimed to test the hypothesis that impairment of cardiac autonomic control relates to increased irregularity in the AF signal. It was found that increased atrial signal irregularity as reflected by SampEn was consistent with an impairment of cardiac autonomic function in both PAF and persistent patients (Sungnoon et al., 2012).

### Entropy for AF mapping

AF mapping is pivotal to catheter ablation, as this helps locate AF triggers and substrates to guide the selection of ablation targets (Baumert et al., 2016). Unfortunately, although ectopic impulses from the pulmonary veins have been shown to initiate AF, catheter ablation of these ectopic foci have only shown limited success in persistent AF cases (Verma et al., 2015). Adding to this limited success is the fact that optimal ablation targets for persistent AF are still debated, as the mechanisms underlying this AF type are not yet well understood (Jalife et al., 2002). As a consequence of this, many approaches to AF mapping have been explored.

Masè et al. explore the use of entropy for quantifying synchronization during atrial fibrillation (Masè et al., 2005). In this study, a synchronization index (*Sy*) was developed using Shannon entropy (ShEn) (Shannon, 1948) to quantify the degree of synchronization during AF. Although AF is often described as being desynchronized, a certain amount of synchronized electrical activity is in fact present, and quantifying this was thought to facilitate the identification of various propagation patterns that may be associated with AF, and hence improve understanding on AF mechanisms and treatment (Masè et al., 2005). *Sy* was defined by quantifying the complexity of the distribution of the time delays between sites using ShEn estimates. Findings from this study showed that a progressive and significant decrease in *Sy* correlated with increasing AF complexity (Masè et al., 2005), using definitions for complexity classes as defined by Wells et al. (1978) (Table 1). *Sy* was also calculated on the whole right atrial chamber, showing the existence of spatial heterogeneities (Masè et al., 2005).

Following this, a number of studies have also utilized entropy for the identification of rotors during AF (Ganesan et al., 2012, 2014; Orozco-Duque et al., 2013; Ugarte et al., 2014). There exists various schools of thought about the mechanisms driving an AF episode, with the rotor theory suggesting that AF is maintained by sites of rotational activation referred to as spiral waves or rotors (Jalife et al., 2002; Waks and Josephson, 2014). There is clinical and experimental evidence to support this theory, and as such, rotors are thought to be potentially effective targets for ablation (Schuessler et al., 1992; Skanes et al., 1998; Vaquero et al., 2008; Narayan et al., 2012). Building on this, Ganesan et al. hypothesized that rotors could be identified through regions of high Shannon entropy (Shannon, 1948), as wavefronts encircling the rotor pivot should result in broadening of the amplitude distribution of bipolar electrograms (EGM) due to their direction-dependent nature (Ganesan et al., 2012). Findings showed that maximum ShEn co-located with the rotor pivot in computer simulated spiral waves, rat and sheep models, and human AF (Ganesan et al., 2012). Ganesan et al. also further explored the characteristics of high ShEn regions at rotor pivot zones (Ganesan et al., 2014) to test the hypothesis that pivot points possess higher ShEn than electrograms recorded at the periphery (Ganesan et al., 2014). It was found that the rotor pivot not only coincided with higher ShEn than those found at the periphery of the spiral wave, but also that pivot zones consistently resulted in maximum ShEn, irrespective of bipolar electrode spacing, signal filtering and rotor meander (Ganesan et al., 2014).

**Figure 6:**
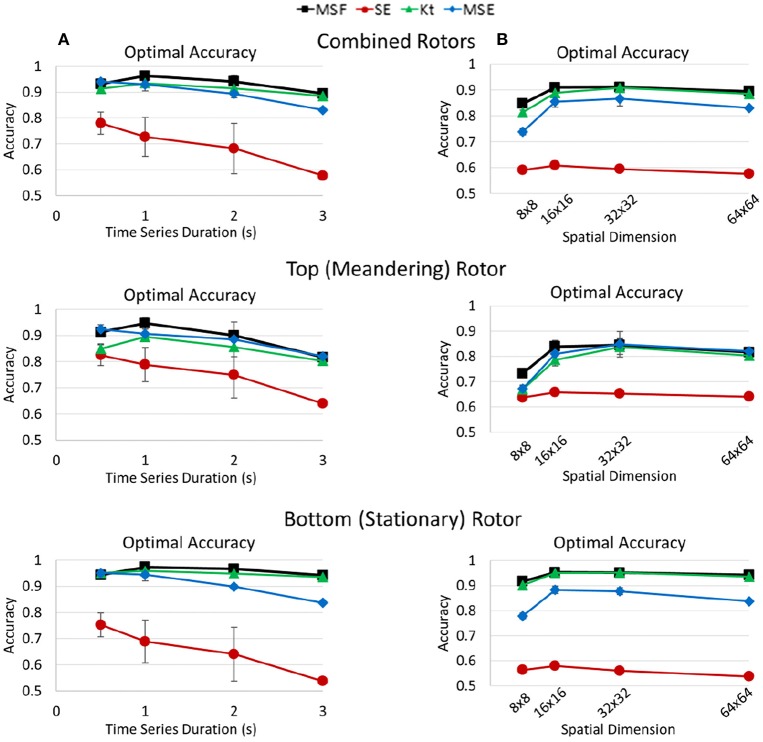
Optimal accuracy of multiscale frequency (MSF), Shannon entropy (SE), kurtosis (Kt), and multiscale entropy (MSE) for the identification of rotors shown in. Optimal accuracies for MSF, SE, Kt, and MSE measures determined by Annoni et al. (2017). The optimal accuracies are shown for both rotors **(Top)**, the top meandering rotor **(Middle)** and bottom stationary rotor **(Bottom)**. Reprinted from Annoni et al. (2017) with permissions.

In a following independent study, Arunachalam et al. supported the ability for ShEn to identify rotors in isolated rabbit hearts (Arunachalam et al., 2015). Specifically, ShEn-based mapping techniques were used to identify pivotal rotor points in optically mapped data acquired from the rabbit hearts, following which the mapping approach was applied to clinical intracardiac human data. Results demonstrated that ShEn could accurately identify the rotor pivot in optically mapped data with known pivot zones (Arunachalam et al., 2015), supporting findings published by Ganesan et al. (2012, 2014). In a more recent study, however, Annoni et al. report that the performance of ShEn is greatly affected by the presence of artifacts, suggesting that other techniques such as multiscale frequency (MSF), Kurtosis (Kt), and Multiscale Entropy (MSE) provide more accurate and robust detection of rotors (Annoni et al., 2017) (Figure 6).

**Figure 7:**
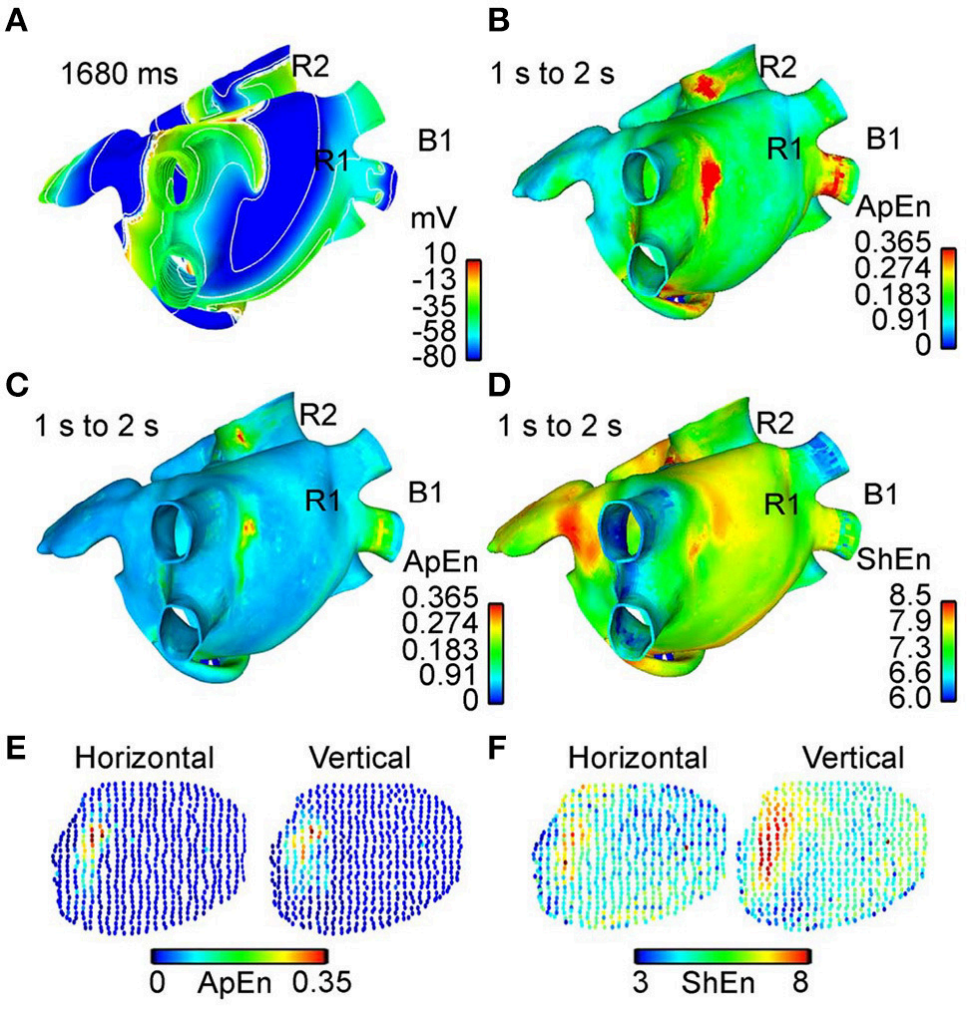
Comparison between different approaches for rotor mapping. 3D maps showing comparisons between different rotor mapping tools as computed by Ugarte et al. (2014). **(A)** Map using the action potential wavefront delimited by contour lines over the 3D atrial model (human) extracted from an interval between 1 and 2 s of simulation. Spinning wavefronts around one point defines the stable rotors R1 and R2. **(B)** Dynamic ApEn map calculated using standard parameters and the unipolar EGM. **(C)** Dynamic ApEn maps using optimized parameters from Ugarte et al. and unipolar EGM. **(D)** ShEn map using unipolar EGM. It can be noted that map **(C)** illustrates better sensitivity for identifying rotor tip. **(E)** Dynamic ApEn map calculated from optimized parameters and the bipolar EGM with vertical and horizontal orientation. The region corresponds to the vicinity of the rotor R1. **(F)** ShEn map using the bipolar EGM obtained from vicinity of R1. Reprinted from Ugarte et al. (2014) with permission.

In a similar vein, Orozco-Duque et al. utilize approximate entropy (ApEn) (Richman and Moorman, 2000) for localizing rotors, under the hypothesis that CFAE are generated by the pivot point of a rotor (Orozco-Duque et al., 2013). Findings suggest that regions of high ApEn also co-located with the rotor pivot (Orozco-Duque et al., 2013). Ugarte et al. also study the relationships between CFAE and the rotor pivot using ApEn (Ugarte et al., 2014) (Figure 7), under the argument that a non-linear dynamic measure will better capture the property of fractionation in comparison to the empirical definitions proposed in the original CFAE study (Nademanee et al., 2004). After simulating AF in a 3D human atrial model, results showed a positive correlation between ApEn and levels of fractionation, suggesting the ability of high ApEn regions to co-locate areas of high fractionation, and in turn the rotor pivot (Ugarte et al., 2014). Sample entropy (SampEn) based approaches have also been explored for this purpose, with Cirugeda-Roldán et al. using SampEn to characterize the degree of fractionation in atrial electrograms (Cirugeda–Roldán et al., 2015). A specificity of 86% and a sensitivity of 77% was reported when discerning between CFAE and non-CFAE electrogram signals (Cirugeda–Roldán et al., 2015).

**Figure 8:**
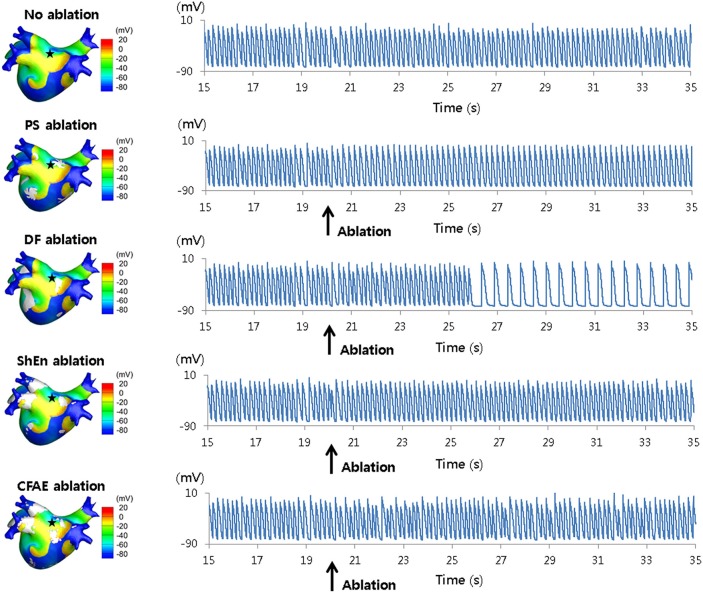
3D voltage maps and action potential curves during virtual ablation using phase singularity (PS), dominant frequency (DF), Shannon entropy (SE), and complex fractionated electrogram (CFAE) approaches. Results from simulated ablation using PS, DF, SE, and CFAE, as performed by Hwang et al. (2016). 3D heart voltage maps are shown on the **(Left)**, with the black star indicating the action potential recording site. Action potential curves are shown on the **(Right)**. Only simulated DF ablation resulted in changes of the action potential, converting AF into atrial tachycardia. Reprinted from Hwang et al. (2016) with permissions.

Investigating the mapping of rotors further, Hwang et al. examined ablation approaches based on Shannon entropy (ShEn) in both 2D and 3D models (Hwang et al., 2016) (Figure 8). The study compared ShEn to other rotor mapping approaches commonly used in literature, namely: phase singularities (PS), dominant frequency (DF), and CFAE cycle length (CFAE-CL) (Hwang et al., 2016). Results from virtual ablation showed that ShEn, PS and CFAE-CL guided approaches did not result in AF termination or modify the AF into slow atrial tachychardia, whilst virtual DF ablation successfully achieved these end-points (Hwang et al., 2016). Additionally, in 2D and 3D *in-silico* models, ShEn was shown to overlap with 33.2 and 27.5% of the rotor tip trajectory respectively, which was outperformed by DF wherein a 71 and 39.7% overlap was seen in the 2D and 3D models respectively (Hwang et al., 2016).

Outside of rotor mapping, entropy can also be used to study causality and information flow. Transfer entropy (TE), which determines the directed exchange of information between two systems (Schreiber, 2000), can be used to investigate the direction and degree of information flow between electrograms. In a study conducted by Kosna et al., TE was used to study information flow between electrograms recorded in the high right atrium (HRA), coronary sinus (CS), and left atrial appendage (LAA) (Kośna et al., 2015). Findings demonstrated that information flow in the heart is symmetric, and that the direction and amount of information flowing between neighboring sites in the atria could be quantified using TE (Kośna et al., 2015). This suggests that studying information flow between different areas of the atria may provide useful insights into the complex wave propagation dynamics during AF.

Recalling the connections between transfer entropy and Granger causality (GC) as discussed previously, work published by Alcaine et al. (2017) uses Granger causality based definitions to develop a multi-variate predictability framework to study information flow and causal relationships between different cardiac sites during AF. Using GC, causal interactions were analyzed between different atrial sites during different rhythms, by considering EGM as stochastic processes that interacts with neighboring atrial sites through information exchange that is driven by atrial activity (Alcaine et al., 2017). Predictability measures were also obtained from the residual variances of linear predictions performed with multivariate autoregressive (MVAR) modeling of involved EGM signals (Alcaine et al., 2017). As such, Alcaine's framework provides a measure of regularity for individual EGMs, in addition to the connectivity between neighboring sites. Using computational simulations and clinical basket catheter data acquired from patients in paroxysmal AF, the study showed that the framework not only allowed different rhythms to be identified (using the regularity measures), but also that the underlying cardiac activity, acquired from simultaneous multi-electrode basket recordings, could be tracked and mapped using GC-based definitions (Alcaine et al., 2017). Although GC is a statistical concept rather than an information theoretic approach, this study demonstrates its connection to transfer entropy and its ability to also study causal relationships.

## Research gaps and potential future developments

Within the past decade, a number of EGM-based quantitative approaches have been developed for AF analysis. These approaches have brought several developments to the study of AF, however, clinical application of these techniques have yet to be achieved due to lack of reproducibility of promising results (Baumert et al., 2016). Underlying this may be the qualitative nature of these approaches, or their need for empirically-based definitions, as well as the lack of understanding of the complex AF mechanism. With this in mind, information theoretic measures may have the potential to provide new insights from study of the statistical properties of signals in AF using a purely quantitative approach.

Thus far, few information theoretic approaches have been explored in the context of atrial fibrillation, particularly in AF mapping applications. Further studies are required to explore the various characteristics of measures such as entropy during AF, and understand their relation to the AF physiology. For example, there is room for future studies to observe the spatial and temporal stability of EGM entropy, as this is an area that has not been investigated thus far. Understanding the spatiotemporal characteristics of AF may determine the presence or absence of spatial and temporal stability, which is important for developing novel adjunctive or primary ablation strategies based on high entropy regions as targets.

In addition to this, the relationships between information theoretic approaches to the micro and anatomical structure of the atrium is also yet to be explored. Anatomical co-registration may further reveal regions with a predisposition to forming rotors or other mechanisms that perpetuate AF, as well as electrical pathways that may be important to AF propagation. Using information theoretic and statistical approaches such as transfer entropy (TE) or Granger causality (GC) to observe information flow between regions of the atria may also help shed light on this, as analysis of connectivity between atrial regions may help infer the wave propagation dynamics of AF, which are highly complex and presently limit the determination of effective ablation targets. Understanding these wave dynamics may again provide potential clinical insight that may lead to more effective ablation strategies.

## Conclusion

Unfortunately, although AF has been a long-standing topic of research, there remains continuing debate regarding the mechanisms underlying the dynamics of the heart rhythm disorder (Schotten et al., 2011). Currently, there is some consensus that AF is the result of an interplay between substrate and triggering mechanism, though it is agreed that this interaction is not yet completely understood, nor is the triggering mechanism responsible.

Due to the complexity in understanding the AF phenomenon, establishing effective mapping approaches have proven hugely difficult, especially for real-time methods that can be used for guided ablation. While direct wavefront mapping during clinical AF procedures would be extremely valuable, current challenges make this approach practically impossible. With this in mind, a logical substitute is to take advantage of the intracardiac electrogram (EGM), which is the primary recording modality currently employed in electrophysiology (EP) clinics. Quantitative analysis of the EGM signal properties using information theoretic approaches has the potential to provide not only a clinically interpretable direct translation to what is seen in practice, but also insights into the system dynamics underlying AF. Aiming to understand the AF dynamics indirectly through analysis of the signal properties is not a left-field approach, as other well studied methods such as CFAE and DF use similar principles. Unlike these techniques however, information theoretic approaches have the benefit of being less reliant on empirically derived definitions.

In summary, while information theory has proved a useful tool for analysis of physiological signals in other fields, it remains underutilized and under-explored in AF studies. As the AF phenomena is far from being understood, understanding the arrhythmia from a signal property perspective and using new approaches may be key to determining effective ablation targets and strategies for the ever increasing AF population.

## Author contributions

DD conducted the literature review, drafted the article, provided critical revision of the article and final approval of the version to be published. LD, AM, PK, and KP provided critical revision of the article and final approval of the version to be published. AG drafted the article, provided critical revision of the article and final approval of the version to be published.

### Conflict of interest statement

The authors declare that the research was conducted in the absence of any commercial or financial relationships that could be construed as a potential conflict of interest.
